# Neural precursor cell delivery induces acute post-ischemic cerebroprotection, but fails to promote long-term stroke recovery in hyperlipidemic mice due to mechanisms that include pro-inflammatory responses associated with brain hemorrhages

**DOI:** 10.1186/s12974-023-02894-8

**Published:** 2023-09-15

**Authors:** Dongpei Yin, Chen Wang, Yachao Qi, Ya-Chao Wang, Nina Hagemann, Ayan Mohamud Yusuf, Egor Dzyubenko, Britta Kaltwasser, Tobias Tertel, Bernd Giebel, Matthias Gunzer, Aurel Popa-Wagner, Thorsten R. Doeppner, Dirk M. Hermann

**Affiliations:** 1 Department of Neurology, University Hospital Essen, University of Duisburg-Essen, Hufelandstr. 55, 45147 Essen, Germany; 2 Institute for Transfusion Medicine, University Hospital Essen, University of Duisburg-Essen, Essen, Germany; 3 Institute for Experimental Immunology and Imaging and Imaging Center Essen (IMCES), University Hospital Essen, University of Duisburg-Essen, Essen, Germany; 4 Institute of Translational Medicine, The First Affiliated Hospital of Shenzhen University, Shenzhen Second People’s Hospital, Shenzhen, China; 5 Leibniz-Institut für Analytische Wissenschaften –ISAS– e.V., Dortmund, Germany; 6 Center of Experimental and Clinical Medicine, University of Medicine and Pharmacy, Craiova, Romania; 7 Department of Neurology, Justus-Liebig University Gießen, Giessen, Germany

**Keywords:** Anti-inflammation, Cholesterol-rich diet, Focal cerebral ischemia, Hypercholesterolemia, Light sheet microscopy, Microglia/macrophage morphology, Middle cerebral artery occlusion, Neuroblast, Neural stem cell, Western diet

## Abstract

**Background:**

The intravenous delivery of adult neural precursor cells (NPC) has shown promising results in enabling cerebroprotection, brain tissue remodeling, and neurological recovery in young, healthy stroke mice. However, the translation of cell-based therapies to clinical settings has encountered challenges. It remained unclear if adult NPCs could induce brain tissue remodeling and recovery in mice with hyperlipidemia, a prevalent vascular risk factor in stroke patients.

**Methods:**

Male mice on a normal (regular) diet or on cholesterol-rich Western diet were exposed to 30 min intraluminal middle cerebral artery occlusion (MCAO). Vehicle or 10^6^ NPCs were intravenously administered immediately after reperfusion, at 3 day and 7 day post-MCAO. Neurological recovery was evaluated using the Clark score, Rotarod and tight rope tests over up to 56 days. Histochemistry and light sheet microscopy were used to examine ischemic injury and brain tissue remodeling. Immunological responses in peripheral blood and brain were analyzed through flow cytometry.

**Results:**

NPC administration reduced infarct volume, blood–brain barrier permeability and the brain infiltration of neutrophils, monocytes, T cells and NK cells in the acute stroke phase in both normolipidemic and hyperlipidemic mice, but increased brain hemorrhage formation and neutrophil, monocyte and CD4^+^ and CD8^+^ T cell counts and activation in the blood of hyperlipidemic mice. While neurological deficits in hyperlipidemic mice were reduced by NPCs at 3 day post-MCAO, NPCs did not improve neurological deficits at later timepoints. Besides, NPCs did not influence microglia/macrophage abundance and activation (assessed by morphology analysis), astroglial scar formation, microvascular length or branching point density (evaluated using light sheet microscopy), long-term neuronal survival or brain atrophy in hyperlipidemic mice.

**Conclusions:**

Intravenously administered NPCs did not have persistent effects on post-ischemic neurological recovery and brain remodeling in hyperlipidemic mice. These findings highlight the necessity of rigorous investigations in vascular risk factor models to fully assess the long-term restorative effects of cell-based therapies. Without comprehensive studies in such models, the clinical potential of cell-based therapies cannot be definitely determined.

**Supplementary Information:**

The online version contains supplementary material available at 10.1186/s12974-023-02894-8.

## Introduction

Despite considerable progress in acute recanalization therapies (i.e., intravenous thrombolysis and/or thrombectomy) [1], ischemic stroke remains the leading cause of disability and second cause of death worldwide [2]. Stroke patients often exhibit long-term neurological deficits, highlighting the need for effective treatments that can promote neurological recovery once a stroke has occurred. While previous efforts focused on enhancing neuronal survival during the acute stroke phase, clinical trials have yielded limited success [3]. As a result, there has been a shift in focus toward the post-acute stroke phase, where substantial efforts are being directed toward promoting brain remodeling and plasticity. Experimental studies in otherwise healthy rodents have demonstrated that cellular therapies can effectively stimulate brain remodeling and neuronal plasticity, leading to improvements in motor-coordination [4, 5]. However, it remains crucial to determine whether similar neurological recovery can be achieved in human patients, who often present with vascular risk factors and co-morbidities that may compromise restorative responses. Unfortunately, recent randomized controlled multicenter trials failed to show the efficacy of restorative treatments in stroke patients [6–8]. Thus, the search for effective therapeutic strategies to enhance neurological recovery in stroke patients remains an ongoing challenge.

Neural stem/precursor cells (NPCs) have emerged as a promising cellular therapy for promoting neurological recovery, brain remodeling, and neuroplasticity following focal cerebral ischemia in rodent models [9–15]. Previous studies have demonstrated the neuroprotective and restorative effects of adult NPCs delivered through various routes (including intravenous, intraarterial, intracerebroventricular, and intraparenchymal administration) over a broad treatment time-window starting from immediately after reperfusion to 28 day post-stroke [16, 17]. Among these delivery routes, intravenous NPC delivery has shown particularly potent recovery-promoting responses, including sustained improvements in motor-coordination, promotion of neuronal survival, restoration of blood–brain barrier integrity, inhibition of brain leukocyte infiltration, reduction in glial scar formation, and prevention of secondary brain atrophy [9, 15–18]. However, thus far, these studies have focused solely on evaluating the effects of NPCs in otherwise healthy ischemic rodents, while the impact of NPCs on neurological recovery and brain remodeling in rodents with vascular risk factors remains unexplored. Therefore, it is essential to investigate how NPCs influence these processes in mice or rats with underlying vascular risk factors.

Hyperlipidemia is a highly prevalent risk factor, which based on American Heart Association guidelines requires therapy in 48.6% of U.S. citizens ≥ 40 years [19]. This condition is characterized by endothelial dysfunction, vascular inflammation, and the formation of atherosclerotic plaques [20]. In the context of focal cerebral ischemia, hyperlipidemic mice fed with a cholesterol-rich Western diet have exhibited increased blood–brain barrier permeability and brain leukocyte infiltration compared with normolipidemic mice on a normal, regular diet [21, 22]. The infiltration of leukocytes, specifically, of polymorphonuclear neutrophils has been implicated in exacerbating ischemic injury in hyperlipidemic mice [22]. However, the effects of intravenously administered NPCs have not been investigated in hyperlipidemic mice. Therefore, this study aims to evaluate the impact of hyperlipidemia on the restorative effects of NPCs by subjecting mice to a cholesterol-rich Western diet for 6 weeks and assessing the effects of adult NPCs on stroke recovery, immunological responses and brain remodeling.

## Materials and methods

### Legal issues, animal housing, randomization and blinding

All experiments were performed with local government approval (Bezirksregierung Düsseldorf) in accordance with EU guidelines (Directive 2010/63/EU) for the care and use of laboratory animals, STAIR, STEPS and ARRIVE guidelines. Experiments were strictly randomized. The experimenters performing the animal experiments and behavioral studies (D.Y., Y.W.) had proven skills in restorative stroke studies. These experimenters were fully blinded at all stages of the study by another researcher (C.W., T.R.D.) preparing the vehicle and NPC solutions. These solutions received dummy names (A and B), which were unblinded after termination of the study. Mice were kept in a regular inverse 12 h:12 h light/dark cycle in groups of 5 animals/cage. Behavioral tests and animal surgeries were always performed in the morning hours throughout the study. Mice had free access to food and drinking water.

### Statistical planning

An a priori sample size calculation was done using an online calculator (https://homepage.univie.ac.at/robin.ristl/samplesize.php?test=ttest). For the central endpoints (e.g., infarct volume, neurological deficits), we postulated that NPCs modified the mean value by 30%, which with an expected standard deviation of 25% of the mean value (Cohen’s D = 1.2) required a sample size of 12 animals/group, given that the alpha error was 5% and the beta error (1–statistical power) was 20%. With a sample size of 10 animals/group, the beta error was 30%, considering that all other variables remained unchanged. In case of neurological tests, we confirmed the negative findings of a first study with once-only NPC administration (that is, delivery immediately after reperfusion) by a second study examining the effects of NPCs administered on three occasions (immediately after reperfusion, 3 and 7 days after MCAO), thus providing proofs that the negative results obtained were not the consequence of by chance observations associated with the beta error.

### Focal cerebral ischemia

Male C57BL6/j mice (Envigo, Indianapolis, IN, U.S.A.) were fed with cholesterol-rich Western diet (E15721-34; Ssniff Spezialdiäten, Soest, Germany) for 6 weeks starting at the age of 3 weeks. We have previously analyzed plasma cholesterol levels following this diet exposure, showing that plasma cholesterol levels were 220.4 ± 58.0 mg/dl in the end of the 6-week period, compared with 56.6 ± 17.4 mg/dl in male wildtype C57BL6/j mice on regular diet [21]. Hyperlipidemic animals exhibited lipid deposits in cerebral microvessels [21]. Focal cerebral ischemia was induced as described before [23, 24]. Briefly, mice were anesthetized with 1.0–1.5% isoflurane (30% O_2_, remainder N_2_O). For analgesia, animals were treated subcutaneously with buprenorphine (0.1 mg/kg b.w.; Reckitt Benckiser, Slough, U.K.). Rectal temperature was maintained by a heating pad system (Fluovac, Harvard apparatus, Holliston, MA, U.S.A.) between 36.5 and 37.0 °C. Cerebral blood flow was monitored by laser Doppler flowmetry using a flexible probe (Perimed, Stockholm, Sweden) attached to the animals’ skulls above the core of the middle cerebral artery territory. A midline neck incision was made. The left common and external carotid arteries were isolated and ligated, and the internal carotid artery was temporarily clipped. A silicon-coated 7.0 nylon monofilament (Doccol, Sharon, MA, U.S.A.) was introduced through a small incision into the common carotid artery and advanced to the carotid bifurcation for MCAO. After 30 min, reperfusion was initiated by monofilament removal. Wounds were carefully sutured. For anti-inflammation, animals received daily i.p. injections of carprofen (4 mg/kg; Bayer Vital, Leverkusen, Germany) during the first 3 day post-stroke. Animals were removed from the study and sacrificed when suffering from central respiratory abnormalities (i.e., apneas) or from severe motor handicaps with inappropriate nurturing, resulting in a weight loss > 20%. The survival rate until the end of the study was 88% in mice sacrificed 48 h post-MCAO, 85% in mice sacrificed 14 day post-MCAO, and 75% in mice sacrificed 56 day post-MCAO.

### NPC culture and transplantation

NPC preparation was performed identically as previously described [13]. Briefly, adult EGFP^+^ NPCs were isolated from the subventricular zone region of 6–8-week-old male enhanced green fluorescence (EGFP)-transgenic mice (C57BL/6-Tg(ACTB-EGFP)1Osb/J; JAX Laboratory, Bar Harbor, ME, U.S.A.). EGFP expression was driven by the actin promoter which allows reliable and stable tracking of grafted NPCs. EGFP^+^ NPCs were grown in serum-free basic Dulbecco’s modified Eagle’s medium (DMEM)/F12 (PAA, Linz, Austria) and supplemented with epidermal growth factor (EGF, 2 µg/ml) and basic fibroblast growth factor (bFGF, 2 µg/ml). To avoid contamination, penicillin–streptomycin (Invitrogen, Frankfurt, Germany) was added to the medium. Cells were incubated with 5% CO_2_ at 37 °C, and growth factors were added every 2–3 days. Passaging of cells was done every 7–10 days and NPCs were used for transplantation from cell passages 3–8.

Following MCAO, mice were randomized into two groups. Via a skin incision, vehicle (200 µl of 0.1 M phosphate-buffered saline [PBS]) or 10^6^ NPCs (dissolved in 200 µl of 0.1 M PBS) were infused through the femoral vein over 10 min immediately after reperfusion onset. The dose of 10^6^ NPCs per mouse was chosen, since it was equivalent to doses in previous studies using normolipidemic mice [9, 13–18]. In defined groups of mice sacrificed at 56 day post-MCAO, additional vehicle or NPC infusions were performed at 3 and 7 day post-MCAO (as above). Infusions were performed in isoflurane anesthesia (as above). In previous studies using normolipidemic mice, NPCs were consistently found to induce long-term cerebroprotection associated with neurological recovery when administered immediately or up to 6 h post-MCAO by our group [13, 14, 16–18].

### Clark score

The Clark score is a comprehensive score, which captures general and focal neurological deficits [25]. It was evaluated at baseline, 3 days, 7 days and then weekly until 56 day post-MCAO.

### Rotarod test

The Rotarod is a motor-coordination test, which consists of a rotating drum (Ugo Basile, model 47600, Comerio, Italy), in which animals are placed, while the drum is accelerating from 4 to 40 rpm. Maximal velocity is achieved after 260 s (maximum testing time 300 s). The time until each animal drops off the drum is measured [23, 24]. Animals were trained three times each on three consecutive days before MCAO. Following a baseline evaluation, animals were tested at 3 days, 7 days and then weekly until 56 day post-MCAO.

### Tight rope test

The tight rope test consists of a 60 cm long rope that is attached to two opposing platforms. Animals are placed on the middle of the rope and the time until reaching one of the platforms is determined (maximum testing time 60 s) [23, 24]. Animals were trained three times on three consecutive days before MCAO. After a baseline examination, animals were tested at 3 days, 7 days and then weekly until 56 day post-MCAO. From the time on the rope and time to platform arrival, tight rope scores were formed as described in Additional file 1: Table S1.

### Animal sacrifice and FITC–albumin gelatin infusion

Mice were deeply anesthetized and transcardially perfused with ice-cold 0.1 M PBS followed by 4% paraformaldehyde in 0.1 M PBS. In animals used for light sheet microscopy, a 0.1% (w/v) solution of gelatin (Sigma-Aldrich, Deisenhofen, Germany) containing FITC-conjugated albumin (Sigma-Aldrich) was subsequently transcardially perfused, as previously reported [26], and the mouse bodies were placed with the head down in ice water over 15 min. All brains were carefully removed and incubated in 4% paraformaldehyde in 0.1 M PBS at 4 °C overnight. Brains used for histochemistry were cut into 20-µm-thick coronal cryostat sections.

### Infarct volume, brain edema and volume, and brain hemorrhage

Coronal sections collected at millimeter intervals were stained with cresyl violet. Sections were scanned and quantified using Image J software (National Institute of Health, Bethesda, MD, U.S.A.). In animals sacrificed at 48 h post-MCAO, infarct volume was measured by subtracting areas of healthy tissue of the ischemic hemisphere from those of the contralesional hemisphere [23]. Brain edema was determined by evaluating the increase of the ipsilateral brain hemisphere volume in comparison with the contralateral brain hemisphere volume [23]. In animals sacrificed at 56 day post-MCAO, striatum volume and whole brain volume were evaluated by analyzing ipsilesional and contralesional brain areas across the forebrain, of which percent volume ratios were determined [23]. At this timepoint, the infarct is completely resolved. Therefore, ischemic injury has converted into brain atrophy. Corpus callosum thickness was evaluated at the bregma level by tracing the corpus callosum area in the ischemic hemisphere from the midline up to one millimeter lateral to the midline [9]. With this procedure, the mean corpus callosum thickness was determined.

For analysis of brain hemorrhages, sections at the level of the bregma were incubated in 0.05% diaminobenzidine substrate kit (Sigma-Aldrich), which is oxidized by erythrocyte peroxidases to produce a dark blue staining [27, 28]. The incidence and area of brain hemorrhages were assessed.

### Immunohistochemistry

Coronal sections from the level of the bregma were immersed in 0.1 M PBS containing 0.1% Triton X-100 (PBS-T) and 10% normal donkey serum (D9663; Sigma-Aldrich). Sections were incubated overnight at 4 °C in Alexa Fluor-594 conjugated polyclonal donkey anti IgG (A-21203; Thermo Fisher Scientific, Waltham, MA, U.S.A.), monoclonal rat anti-CD31 (550274; BD Biosciences, Heidelberg, Germany), polyclonal goat anti-intercellular adhesion molecule (ICAM-1) (AF796; R&D Systems, Minneapolis, MN, U.S.A.), polyclonal rabbit anti-collagen-IV (AB756P; Merck-Millipore, Darmstadt, Germany), monoclonal rat anti-glycoprotein-Ibα (GP-Ibα) (M043-0; Emfret Analytics, Eibelstadt, Germany), polyclonal rabbit anti-ionized calcium binding adaptor protein (Iba)-1 (019–19741; Wako Chemicals, Neuss, Germany), monoclonal rat anti-glial fibrillary acidic protein (GFAP) (130300; Thermo Fisher Scientific, Waltham, MA, U.S.A.), or monoclonal rabbit anti-neuronal nuclei (NeuN) (ab177487; Abcam, Cambridge, U.K.) antibodies, which were detected with fluorescent-labeled secondary antibodies. Labelings were counterstained with Hoechst-33342 (62249; Thermo Fisher Scientific, Waltham, MA, U.S.A). Sections were evaluated under a Zeiss AxioObserver.Z1 inverted epifluorescence microscope equipped with Apotome optical sectioning by evaluating integrated signal intensities in the striatum (extravasated IgG, ICAM-1, GFAP), by analyzing the microvessel length per mm^2^ (CD31), by counting the number of microvessel profiles exhibiting platelet microclots per mm^2^ (collagen-IV/GP-Ibα) or by counting the number of cells per mm^2^ (Iba-1) or in the striatum (NeuN). Optical sectioning was used for correcting cell overcounts.

### Microglia/macrophage morphology analysis

In representative regions of interest (ROIs) in the ischemic striatum, confocal microscopy was performed using the Leica SP8 confocal microscope (objective HC PL APO CS2 63x/1.30, Leica Microsystems, Wetzlar, Germany) to evaluate the three-dimensional morphology of Iba-1^+^ microglia/macrophages. Z-stacks measuring 184.52 × 184.52 × 15 µm were obtained at 0.5 µm interslice distance. To analyze the 3D morphology of Iba-1^+^ cells, background and artefacts were supressed with standard ImageJ tools using fully automated scripts to avoid experimenter bias. The analysis of 3D cell morphology was conducted using the 3DMorph software, as previously described [29]. In brief, the cell objects were detected by automated threshold setting and segmentation. After skeletonization, morphological metrics defining average branch length, cell territory, cell volume and ramification index were derived. To our knowledge, this is the first workflow that provides the opportunity to discriminate single microglia/macrophage cells in densely packed brain lesions, allowing for the precise quantification of cell morphology.

### Light sheet imaging and image processing

For clearing mouse brains perfused with FITC–albumin hydrogel, we used an established tetrahydrofuran (THF; Sigma-Aldrich)/ethyl cinnamate (ECi; Sigma-Aldrich) protocol [26]. Cleared whole mouse brains were imaged using an Ultramicroscope 2 (LaVision BioTec, Bielefeld, Germany) microscope equipped with bidirectional light sheet illumination, and an Andor Neo sCMOS camera having a 2560 × 2160 chip of 6.5 μm pixel size. We performed serial optical imaging of the brains in a ventral–dorsal direction by exciting the FITC–albumin labeled vessels using a 488 nm diode laser and a 525/50 nm bandpass emission filter. Images were acquired at 6.4× magnification in the axial direction at 2 μm steps. For image rendering, Bitplane software (Imaris, Cologne, Germany) was used. For detailed vascular quantification, stacks of 501 images were acquired in ischemic brain tissue with 6.4× magnification and a step size of 2 µm in a ventro-dorsal direction starting at bregma − 6.24 mm and ending at bregma − 4.26 mm. From each of stack, ROIs measuring 508 × 508 × 1000 µm and 305 × 305 × 600 µm, respectively, were chosen in the dorsolateral striatum and parietal cortex, as previously reported [30]. ROIs in the parietal cortex were adjacent to the most outer corners of the striatal ROIs, with the external capsule in between.

Following image preprocessing, the cerebral microvasculature was analyzed using the VesselExpress pipeline [26]. VesselExpress is an automated open-source pipeline that integrates image segmentation, skeletonization and graph analysis, and has been validated for medium and high throughput processing due to high degree of automation, robustness, and parallelized data processing [26]. Using VesselExpress, we determined a comprehensive set of network characteristics, which included (a) microvascular length density (i.e., the total vessel length per brain volume), (b) branching point density (i.e., the total number of branching points per brain volume), (c) microvascular volume density (i.e., the total vessel volume per brain volume), (d) mean branch length between two branching points, and (e) mean branch diameter.

### Flow cytometry of leukocytes

Flow cytometry analysis of blood and brain samples was performed as described before [25]. In brief, blood samples were taken from the animals’ hearts at 48 h post-MCAO, and ischemic brain hemispheres were harvested from the same animals, which were dissociated for further use. After erythrocyte lysis followed by two washing steps, single cell suspensions were stained for 30 min at 4 °C using antibody cocktails listed in Additional file 1: Table S2. Cell suspensions were subsequently analyzed on a FACS Aria III flow cytometer (BD Biosciences) using FlowJo software V10 (Ashland, OR, U.S.A.). The gating strategy is summarized in Additional file 1: Fig. S1. Leukocyte numbers were quantified on a Cytoflex flow cytometer (Beckman–Coulter).

### Statistical data analysis

Statistical analysis was performed using GraphPad Prism version 8.0.2 for Windows software (GraphPad Software, San Diego, California U.S.A.). LDF recordings, body weight, neurological deficits and behavioral tests were analyzed by repeated measurement ANOVA followed by LSD posthoc tests. Histochemical data, which were normally distributed, were evaluated by two-way ANOVA followed by LSD posthoc tests (comparisons between ≥ 3 groups) or by unpaired two-tailed *t* tests (comparisons between 2 groups). Non-normally distributed data were evaluated by Kruskal–Wallis test followed by Dunn’s tests. Hemorrhage incidence data were assessed by chi^2^ tests. Data involving repeated measurements were presented as mean ± S.D. values, all other data as median (mean) ± interquartile ranges (IQR) with minimum and maximum data as whiskers. Incidence data were reported as percentage rates. *P* values < 0.05 were defined to indicate statistical significance.

## Results

### Adult NPCs induce cerebroprotection and reduce blood–brain barrier permeability in the acute stroke phase in normolipidemic and hyperlipidemic mice

**Fig. 1:**
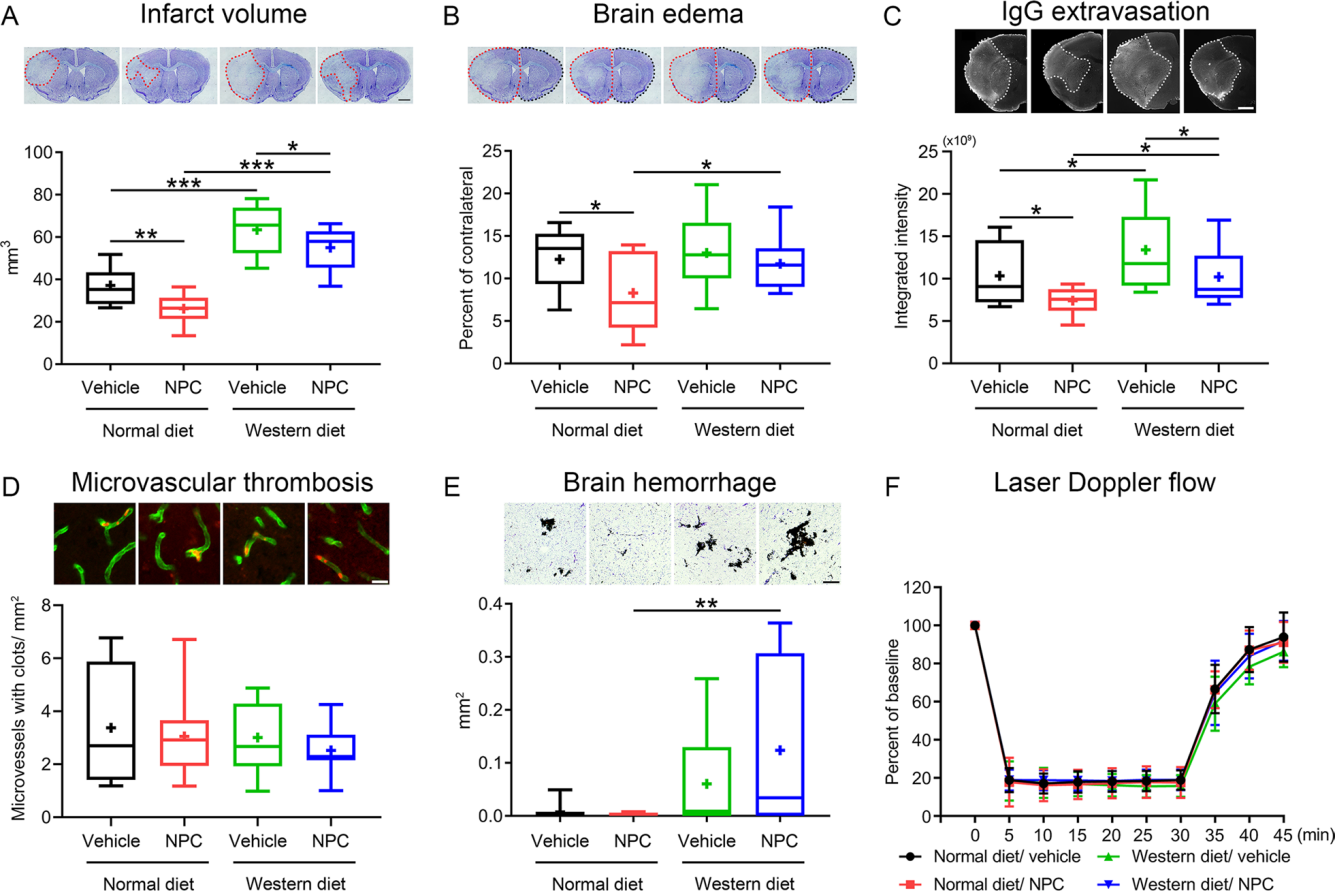
Neural precursor cells (NPCs) induce cerebroprotection and reduce blood–brain barrier (BBB) permeability in the acute stroke phase in normolipidemic and hyperlipidemic mice. **A** Infarct volume and **B** brain edema evaluated by cresyl violet staining, **C** extravasated serum IgG abundance and **D** collagen-IV^+^ ischemic microvessels exhibiting glycoprotein-Ibα (GP-Ibα)^+^ microthrombosis, assessed by immunohistochemistry, **E** brain hemorrhage formation, determined by diaminobenzidine staining, as well as **F** laser Doppler flow during ischemia and after reperfusion above the core of the middle cerebral artery territory of normolipidemic mice on normal diet and hyperlipidemic mice on Western diet, which were exposed to 30 min intraluminal middle cerebral artery occlusion (MCAO). Mice were intravenously treated with vehicle (200 µl of 0.1 M phosphate-buffered saline [PBS]) or adult NPCs (10^6^ cells in 200 µl of 0.1 M PBS) immediately after reperfusion, followed by animal sacrifice at 48 h post-MCAO. Representative brain sections and microphotographs are shown. Note that hyperlipidemia exacerbates infarct volume, BBB permeability, and brain hemorrhage formation, but does not abolish the neuroprotective effects of NPCs in the acute stroke phase. Furthermore, note that GP-Ibα^+^ microthrombosis is not influenced by NPCs in either group, whereas brain hemorrhage formation is increased in NPC-treated hyperlipidemic mice compared with NPC-treated normolipidemic mice. Data are medians (lines inside boxes)/means (crosses inside boxes) ± interquartile ranges with minimum/maximum values as whiskers [in (**A**–**E**)] or means ± S.D. values [in (**F**)]. *P < 0.05/**P < 0.01/***P < 0.001 (*n* = 12 mice for normal diet/vehicle, *n* = 12 for normal diet/NPC, *n* = 10 for Western diet/vehicle, *n* = 11 for Western diet/NPC). Scale bar, 1 mm [in (**A–C**)], 20 µm [in (**D**)] and 200 µm [in (**E**)]

Intraluminal MCAO induced reproducible brain infarcts in normolipidemic mice, which involved the striatum and overlying cerebral cortex (Fig. 1A) and were associated with moderate brain edema (Fig. 1B) and IgG extravasation (Fig. 1C) at 48 h. In line with earlier studies from our group examining NPC administration immediately after reperfusion [14, 16], infarct volume, brain edema and IgG extravasation were significantly reduced by intravenous NPC delivery (Fig. 1A–C). Infarct volume and IgG extravasation were increased in hyperlipidemic mice on Western diet (Fig. 1A, C). NPC delivery significantly decreased infarct volume and IgG extravasation in hyperlipidemic mice (Fig. 1A, C).

### Adult NPCs do not influence microvascular ICAM-1 abundance and microvascular thrombosis post-ischemia in either normolipidemic or hyperlipidemic mice

ICAM-1 is an adhesion molecule, which is expressed on cerebral endothelial cells mediating the brain invasion of leukocytes, for which we have previously shown that it was downregulated upon mesenchymal stromal cell (MSC)-derived extracellular vesicle delivery in MCAO mice [25, 31]. Hence, we asked if ICAM-1 abundance was increased by hyperlipidemia and if it was reduced by intravenous NPC administration. Notably, ICAM-1 abundance, assessed by immunohistochemistry, did not differ between normolipidemic mice on normal diet and hyperlipidemic mice on Western diet, and it was not influenced by NPC delivery (Additional file 1: Fig. S2A).

Microvascular thrombosis triggers ischemic injury in MCAO mice [32] and has been shown to be responsible for the exacerbated ischemic injury in mice exposed to lipopolysaccharide-induced sepsis [33]. We therefore examined if microvascular thrombosis was influenced by hyperlipidemia and intravenous NPC delivery. Microvascular thrombosis, evaluated by GP-Ibα immunohistochemistry, did not differ between mice on normal diet and mice on Western diet, and it was not altered by NPC administration (Fig. 1D).

### Brain hemorrhage formation is increased in the ischemic brains of NPC-treated hyperlipidemic mice

In rats with streptozotocin-induced type-1 diabetes, administration of mesenchymal stromal cells has previously been shown to increase the incidence of brain hemorrhages post-MCAO, which was associated with poor stroke outcomes [34]. Thus, we investigated how adult NPC delivery influenced brain hemorrhage formation in normolipidemic and hyperlipidemic MCAO mice. In normolipidemic mice, few hemorrhages were noted in the ischemic brain tissue, which were not influenced by NPCs (Fig. 1E; Additional file 1: Fig. S2B). Brain bleedings were increased in hyperlipidemic compared with normolipidemic MCAO mice, and further elevated by NPCs (Fig. 1E). Both the incidence and area of brain hemorrhages were significantly higher in NPC-treated hyperlipidemic compared with NPC-treated normolipidemic mice (Fig. 1E; Additional file 1: Fig. S2B).

Considering that reperfusion predisposes to brain hemorrhage, we also studied, if the severity of ischemia or reperfusion differed in response to NPC treatment in normolipidemic and hyperlipidemic mice. Intraluminal MCAO induced reproducible ischemia in the vascular territory of the middle cerebral artery, as indicated by laser Doppler flow (LDF) recordings, which was followed by stable reperfusion to close to baseline levels after monofilament removal (Fig. 1F). LDF recordings did not differ in normolipidemic and hyperlipidemic mice, and they were not influenced by NPCs (Fig. 1F).

### NPCs do not influence microglia/macrophage abundance or activation in the acute stroke phase assessed by morphological analysis

**Fig. 2:**
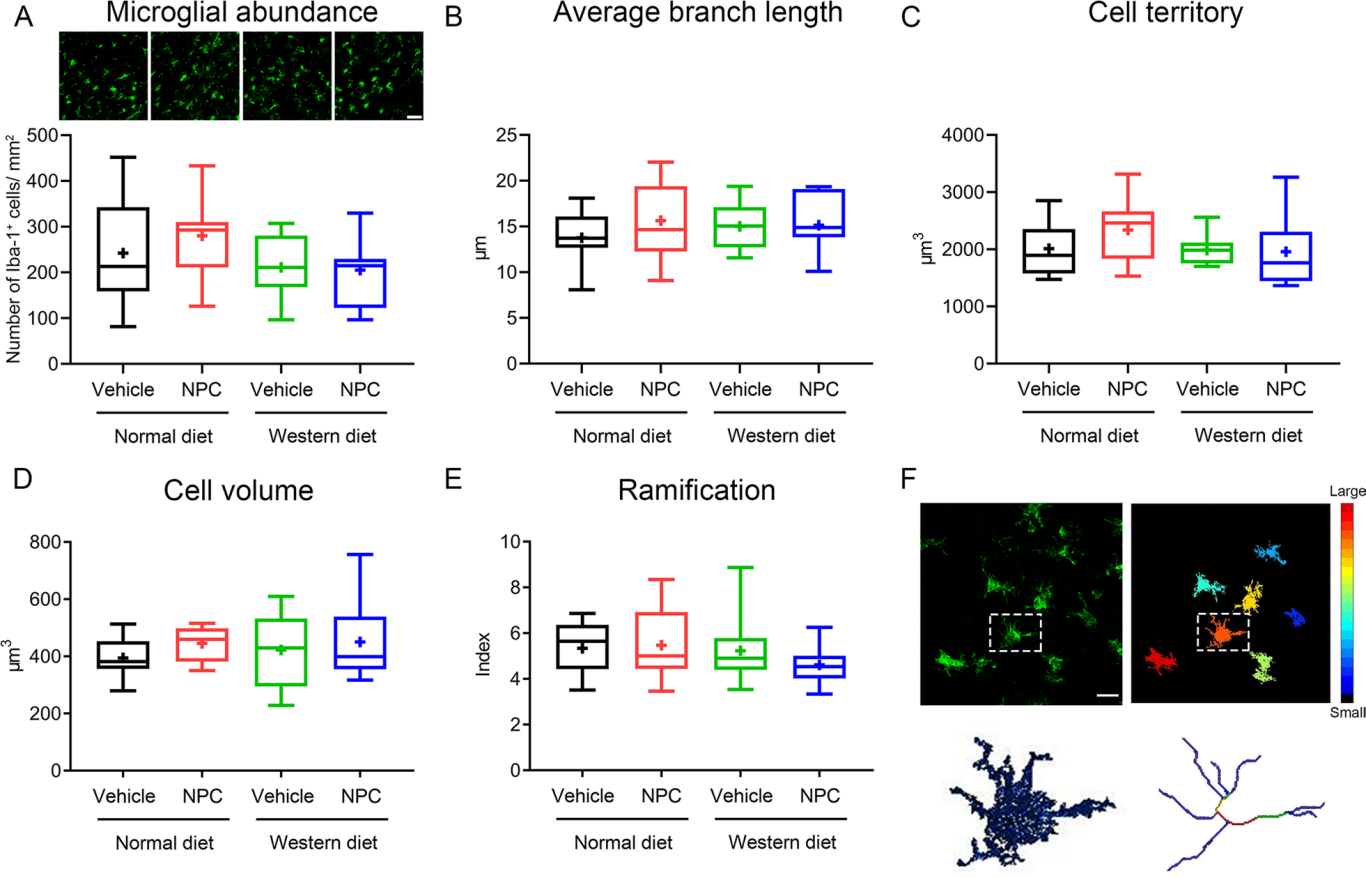
NPC administration does not influence microglia/macrophage abundance and activation in the acute stroke phase in normolipidemic and hyperlipidemic mice. **A** Microglia/macrophage abundance, evaluated by confocal microscopy, **B** average branch length, **C** cell territory, **D** cell volume, and **E** ramification index as assessed of Iba-1 immunohistochemistry in the ischemic striatum of mice on normal diet or cholesterol-rich Western diet exposed to MCAO, which were intravenously treated with vehicle or adult NPCs immediately after reperfusion (as before), followed by animal sacrifice at 48 h post-MCAO. **F** Morphological analysis was done in representative high-resolution 3D stacks, in which single microglia/macrophage cells were reconstructed, segmented and skeletonized (example highlighted in square). Data are medians (lines inside boxes)/means (crosses inside boxes) ± interquartile ranges with minimum/maximum values as whiskers. No significant group differences were noted (*n* = 12 mice for normal diet/vehicle, *n* = 12 for normal diet/NPC, *n* = 10 for Western diet/vehicle, *n* = 11 for Western diet/NPC). Scale bar, 50 μm [in (**A**)] and 20 μm [in (**F**)]

We have previously found that NPC delivery reduces the accumulation of microglia/macrophage cells in periinfarct areas at post-acute timepoints (6–84 days post-ischemia) in normolipidemic MCAO mice [14, 16]. We therefore asked if NPC delivery modified microglia/macrophage abundance and activation, which might have contributed to altered ischemic injury development. Densitometric and morphological analysis of high-resolution images revealed that Iba-1^+^ microglia/macrophage number, average branch length, cell territory, cell volume, and cell ramification were not influenced by NPCs at 48 h post-MCAO (Fig. 2A–E), as shown in 3D stacks, in which single cells were reconstructed, segmented, and skeletonized for quantitative analysis (Fig. 2F).

### NPCs reduce brain leukocyte infiltrates in the ischemic brain of hyperlipidemic mice

**Fig. 3:**
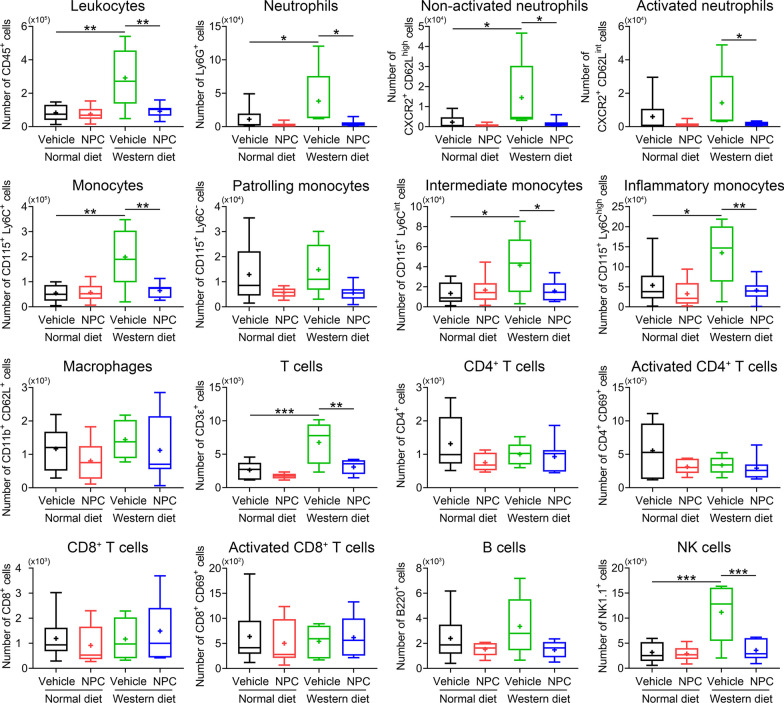
NPC delivery reduces leukocyte infiltration in the ischemic brain of normolipidemic and hyperlipidemic mice. Total counts and activation states of leukocytes and leukocyte subsets in the brain of mice on normal diet or cholesterol-rich Western diet exposed to MCAO, which were intravenously treated with vehicle or NPCs immediately after reperfusion (as before), followed by animal sacrifice at 48 h. Note the pronounced reduction of myeloid cells (neutrophils, monocytes) and lymphoid cells (CD4^+^ T cells, NK cells) by NPCs in the ischemic brain of mice on Western diet, in which inflammatory responses were markedly increased compared with mice on normal diet. Data are medians (lines inside boxes)/means (crosses inside boxes) ± interquartile ranges with minimum/maximum values as whiskers. *P < 0.05/**P < 0.01/***P < 0.001 (*n* = 6 mice for normal diet/vehicle, *n* = 6 for normal diet/NPC, *n* = 5 for Western diet/vehicle, *n* = 7 for Western diet/NPC)

Brain-invading leukocytes and, specifically, polymorphonuclear neutrophils contribute to ischemic brain injury after intraluminal MCAO [35]. Under conditions of hyperlipidemia induced by Western diet, brain-invading leukocytes and, more specifically, neutrophils, mediated the exacerbated ischemic injury of hyperlipidemic compared with normolipidemic mice [22]. We thus asked, how NPC delivery influenced brain leukocyte infiltrates in MCAO mice on Western diet. By applying flow cytometry we found that the number of brain infiltrating CD45^+^ leukocytes, neutrophils (both activated and non-activated), monocytes (including inflammatory monocytes), T cells and NK cells were significantly higher in the ischemic brain of mice on Western diet than mice on normal diet (Fig. 3) [22]. NPC delivery significantly reduced the number of infiltrating CD45^+^ leukocytes, namely of neutrophils (both activated and non-activated), monocytes (including inflammatory monocytes), T cells and NK cells in the brain of Western diet MCAO mice (Fig. 3).

### NPCs increase blood leukocyte counts in hyperlipidemic ischemic mice

**Fig. 4:**
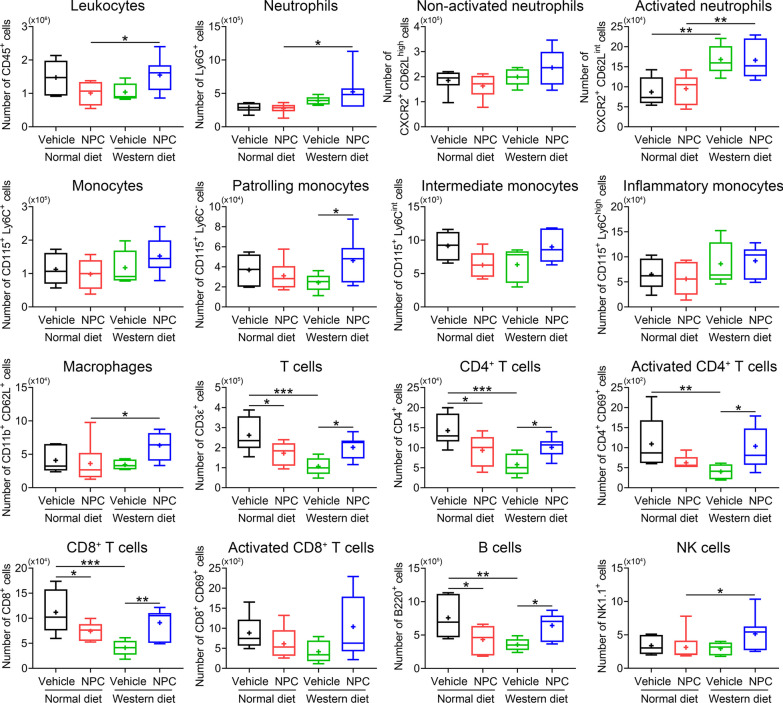
NPC administration increases inflammatory responses in the blood of hyperlipidemic mice but reduces inflammatory responses of normolipidemic mice. Total counts and activation states of leukocytes and leukocyte subsets in the blood of MCAO mice on normal diet or cholesterol-rich Western diet, which were intravenously treated with vehicle or NPCs immediately after reperfusion (as before), followed by animal sacrifice at 48 h. Note the increased counts of neutrophils, activated neutrophils, patrolling monocytes, CD4^+^ and CD8^+^ T cells, activated CD4^+^ T cells, B cells and NK cells in the blood of Western diet mice on NPCs. Data are medians (lines inside boxes)/means (crosses inside boxes) ± interquartile ranges with minimum/maximum values as whiskers. *P < 0.05/**P < 0.01/***P < 0.001 (*n* = 6 mice for normal diet/vehicle, *n* = 6 for normal diet/NPC, *n* = 5 for Western diet/vehicle, *n* = 7 for Western diet/NPC)

In young and aged MCAO mice, we previously found that anti-inflammatory effects of MSC-derived extracellular vesicles in the brain closely went in line with anti-inflammatory effects in peripheral blood, which promoted us to postulate a peripheral mode of action of this cellular therapy [25, 31]. Hence, we asked, if intravenously administered NPCs also conferred anti-inflammation in the blood of MCAO mice. Contrary to this hypothesis, we found that NPC delivery moderately influenced blood leukocytes in normolipidemic MCAO mice. Thus, total leukocyte, neutrophil (both activated and non-activated), monocyte, macrophage and NK cell counts were unchanged, whereas CD4^+^ and CD8^+^ T cells and B cells were reduced upon NPC delivery (Fig. 4). This pattern markedly differed in hyperlipidemic mice. Thus, NPCs increased leukocyte, patrolling monocyte, macrophage, CD4^+^ and CD8^+^ T cell, B cell and NK cell counts in MCAO mice on Western diet (Fig. 4), indicating a pro-inflammatory effect that differed from the pattern in normolipidemic mice.

### NPCs do not influence ischemic injury, microvascular length density or branching point density in the subacute stroke phase in hyperlipidemic mice

**Fig. 5:**
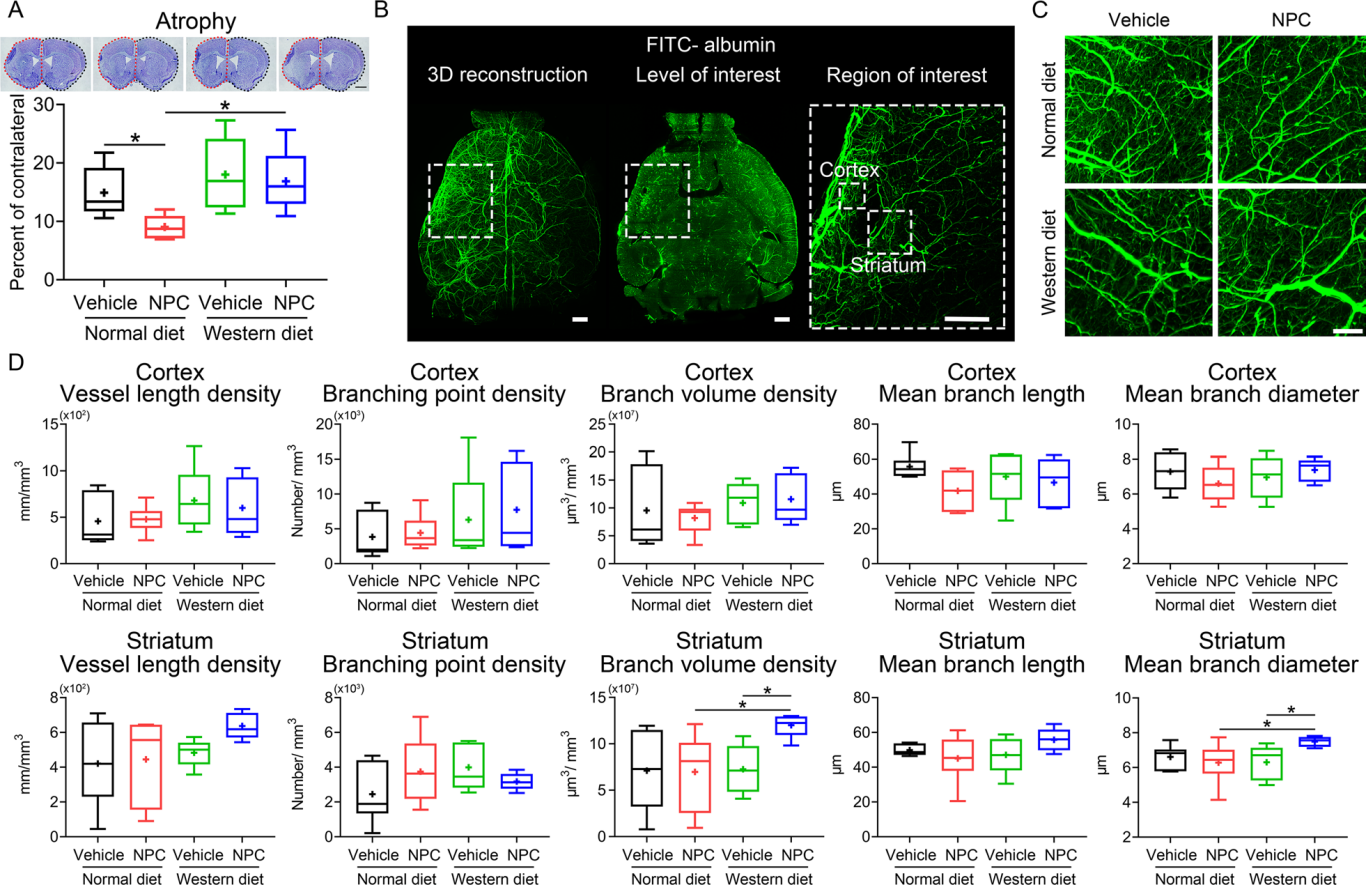
NPC delivery does not influence brain atrophy in the sub-acute stroke phase in hyperlipidemic mice but moderately increases microvascular branch volume and mean branch diameter in the lesion core. **A** Brain atrophy evaluated by cresyl violet staining of MCAO mice on normal diet or cholesterol-rich Western diet, which were intravenously treated with vehicle or adult NPCs immediately after reperfusion (as before), followed by animal sacrifice after 14 days. **B**, **C** 3D light sheet fluorescence microscopy (LSFM) of cerebral microvessels of the same mice, which were intravenously labeled with FITC–albumin hydrogel immediately before animal sacrifice followed by brain clearing and imaging, as outlined in the Materials and methods section. Representative axial overview images with magnification images depicting the regions of interest in the ischemic striatum and cortex [in (**B**)] and representative maximum intensity projection images in the ischemic striatum for the four groups [in (**C**)] are shown. **D** Quantitative analysis of microvascular length density, branching point density, branch volume, mean branch length, and mean branch diameter evaluated by 3D LSFM in the ischemic cortex and striatum of MCAO mice on normal diet and Western diet, which were treated with vehicle or NPCs. Data are medians (lines inside boxes)/means (crosses inside boxes) ± interquartile ranges with minimum/maximum values as whiskers. *P < 0.05 (*n* = 6 mice for normal diet/vehicle, *n* = 6 for normal diet/NPC, *n* = 5 for Western diet/vehicle, *n* = 5 for Western diet/NPC). Scale bar, 1 mm [in (**A**)], 500 μm [in (**B**)] and 100 μm [in (**C**)]

In view that NPCs conferred cerebroprotection in the acute stroke phase in normolipidemic and hyperlipidemic mice, we asked if this effect was maintained in the subacute stroke phase, at 14 day post-MCAO. Analysis of brain atrophy, a marker of long-term injury, by cresyl violet staining revealed that NPC administration reduced brain atrophy in normolipidemic, but not in hyperlipidemic MCAO mice (Fig. 5A).

Microvascular remodeling and angiogenesis are important determinants of neurological recovery in response to restorative stroke treatments [5], but it has previously been shown that MSC delivery induces maladaptive angiogenesis associated with increased BBB permeability in type-1 diabetic MCAO mice [34]. Hence, we asked if NPCs compromised microvascular remodeling in the ischemic brain of hyperlipidemic mice. Analysis of a comprehensive set of microvascular network characteristics by 3D LSFM revealed that NPC delivery did not influence microvascular length density, branching point density and mean branch length in the ischemic cortex or striatum of MCAO mice on normal diet or Western diet (Fig. 5B–D). Yet, NPCs increased branch volume density and mean branch diameter in the ischemic striatum of Western diet mice (Fig. 5D). Hence, NPCs altered the remodeling of microvessels post-ischemia in hyperlipidemic mouse brains.

### NPCs do not influence motor-coordination recovery in the post-acute stroke phase in hyperlipidemic mice

**Fig. 6:**
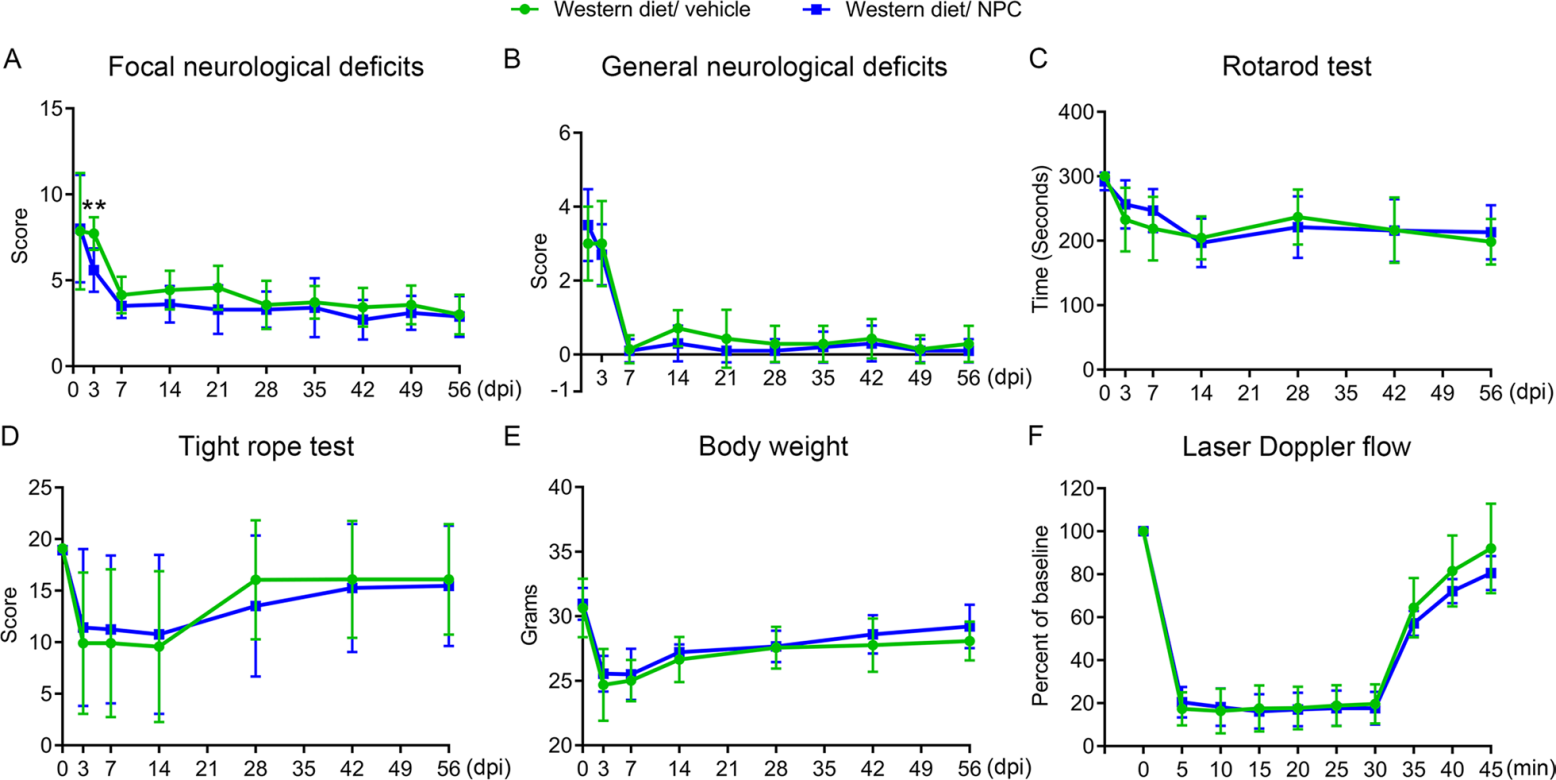
NPC administration mitigates neurological deficits in the acute, but not the post-acute stroke phase in hyperlipidemic mice. **A** Focal neurological deficits and **B** general neurological deficits in the Clark score, motor-coordination performance in **C** the Rotarod test and **D** the tight rope test, **E** body weight and **F** laser Doppler flow above the core of the vascular territory of the middle cerebral artery of MCAO mice on Western diet, which were intravenously treated with vehicle or NPCs immediately, at 3 and 7 days after reperfusion (three times, dosing as before), followed by animal sacrifice after 56 days. Note the improvement of focal neurological deficits in the Clark score by NPCs at 3 days [see (**B**)], which disappeared at later timepoints. Note the absence of changes in motor-coordination tests. For mice treated once only with NPCs see Suppl. Figure 3. Data are means ± S.D. values. **P < 0.01 (*n* = 7 mice for Western diet/vehicle, *n* = 10 for Western diet/NPC)

Considering that cerebroprotection by intravenously administered NPCs was noted in the acute stroke phase, but not sub-acute stroke phase, we asked how NPC delivery influenced motor-coordination recovery in hyperlipidemic MCAO mice. We had previously shown that NPC delivery enhanced motor-coordination recovery in normolipidemic mice when administered immediately after reperfusion and up to 72 h after MCAO [13, 14, 16–18]. In a first set of mice, we administered NPCs immediately after reperfusion onset in line with two earlier studies of our group revealing recovery-promoting effect of adult NPCs [14, 16] and examined motor-coordination recovery by Rotarod and tight rope tests over up to 56 day post-MCAO. In this study, NPC administration did not influence motor-coordination recovery as revealed by Rotarod and tight rope tests (Additional file 1: Fig. S3A, B). In view of a possible beta error, also considering conceivable dose-dependent actions of NPCs, we repeated this study, now administering 3 consecutive NPC infusions immediately after reperfusion, after 3 days and 7 days. In this study, NPCs improved focal neurological deficits evaluated by the Clark score at 3 days (Fig. 6A), but again did not influence focal or general neurological deficits at later timepoints (Fig. 6A, B) or motor-coordination performance assessed by Rotarod and tight rope tests at any timepoint (Fig. 6C, D). NPCs did not influence body weight (Additional file 1: Fig. S3C, Fig. 6E) or laser Doppler flow recordings (Additional file 1: Fig. S3D, Fig. 6F) at any timepoint in both studies.

### NPCs do not influence long-term brain tissue survival and remodeling in the post-acute stroke phase

**Fig. 7:**
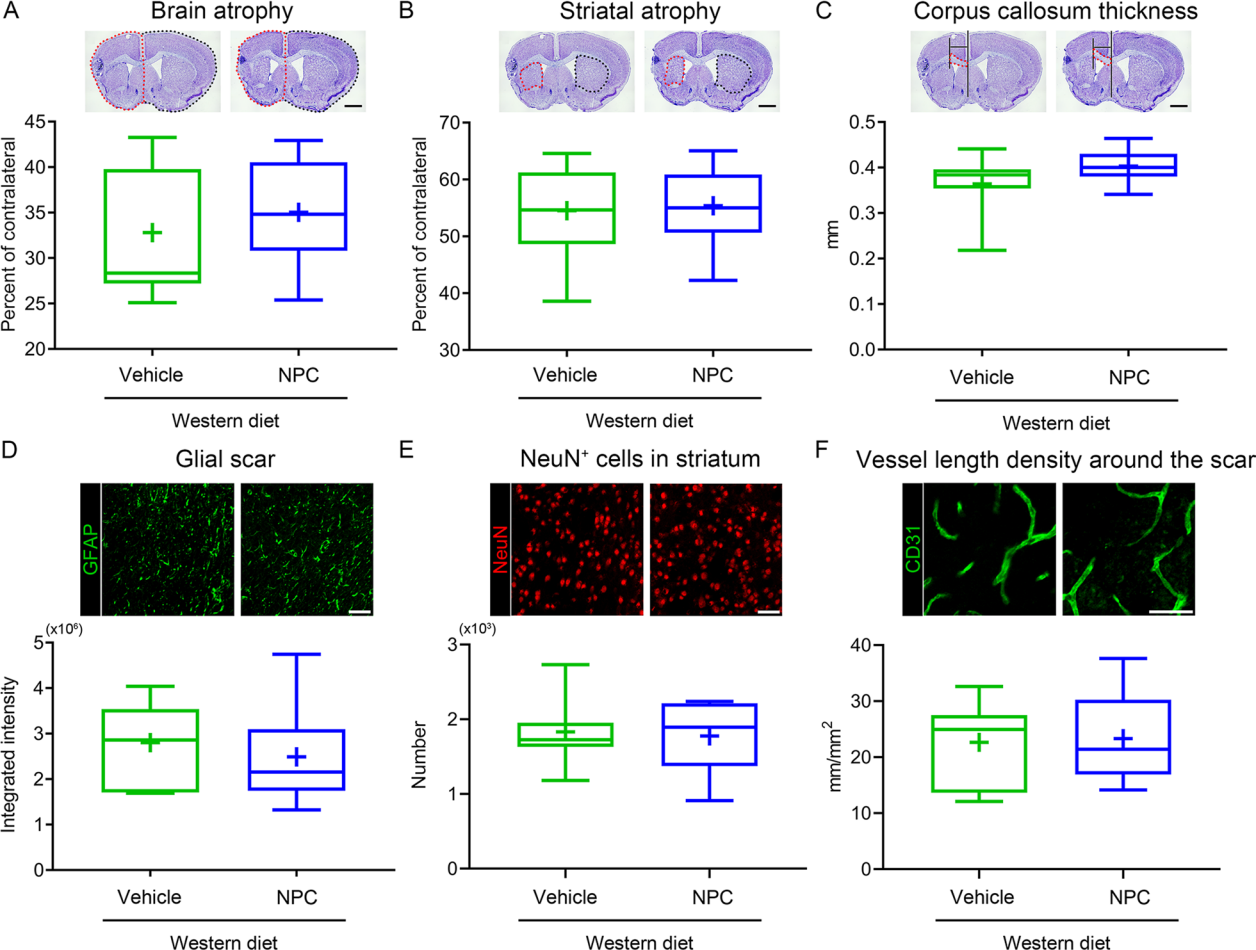
NPC delivery does not influence brain atrophy, glial scar formation, long-term neuronal survival, and microvessel density in the chronic stroke phase in hyperlipidemic mice. **A** Brain atrophy, **B** striatal atrophy and **C** corpus callosum thickness assessed by cresyl violet staining, **D** glial fibrillary acidic protein (GFAP) expression, **E** neuronal nuclei (NeuN)^+^ neuronal number in the striatum and **F** density of CD31^+^ cerebral microvessels in the striatum assessed by immunohistochemistry in MCAO mice on Western diet, which were intravenously treated with vehicle or NPCs immediately after reperfusion, at 3 and 7 days (three times, dosing as before), followed by animal sacrifice after 56 days. For mice treated once only with NPCs see Additional file 1: Fig. S4. Data are medians (lines inside boxes)/means (crosses inside boxes) ± interquartile ranges with minimum/maximum values as whiskers. No significant group differences were noted (*n* = 7 mice for Western diet/vehicle, *n* = 10 for Western diet/NPC). Scale bar, 1 mm [in (**A**–**C**)] and 50 μm [in (**D**–**F**)]

Substantial striatal atrophy and, to lesser extent, whole brain atrophy were noted in the brains of vehicle-treated mice on Western diet, as shown in cresyl violet stainings at 56 day post-MCAO (Additional file 1: Fig. S4A, B, Fig. 7A, B). To evaluate possible structural cerebroprotective effects of NPCs in the absence of effects on long-term neurological recovery, we examined the effects of NPCs on striatal and whole brain atrophy, as well as on the thickness of the corpus callosum. NPC delivery did not influence striatal atrophy, whole brain atrophy and corpus callosum thickness of hyperlipidemic MCAO mice (Additional file 1: Fig. S4A–C, Fig. 7A–C). Similarly, NPCs did not alter long-term astroglial scar formation (Additional file 1: Fig. S4D, Fig. 7D), neuronal survival (Fig. 7E) and microvascular length density (Fig. 7F), evaluated by GFAP, NeuN, and CD31 immunohistochemistry, respectively, in the periinfarct tissue of hyperlipidemic MCAO mice.

## Discussion

We herein showed that adult NPC delivery immediately post-MCAO reduced infarct volume, blood–brain barrier permeability and neurological deficits in the acute stroke phase in normolipidemic and hyperlipidemic mice, but did not promote brain parenchymal remodeling associated with long-term neurological recovery in the post-acute stroke phase in hyperlipidemic mice. The brain infiltration of a broad set of myeloid (neutrophils, monocytes) and lymphoid (T cells, NK cells) leukocytes was reduced by NPC administration in both normolipidemic and hyperlipidemic MCAO mice, while blood leukocyte (namely, neutrophil, monocyte, CD4^+^ and CD8^+^ T cell) counts and activation were increased by NPCs in hyperlipidemic MCAO mice. In hyperlipidemic mice, NPCs did not influence peri-infarct endothelial ICAM-1 abundance, microvascular thrombosis, microglia/macrophage abundance or activation (the latter assessed by morphological analysis), microvascular length density or branching point density (evaluated using light sheet microscopy), astroglial scar formation, long-term neuronal survival or brain atrophy at timepoints up to 56 day post-MCAO. The incidence and area of brain hemorrhages was increased in the ischemic brains of NPC-treated hyperlipidemic compared with NPC-treated normolipidemic MCAO mice.

The NPC treatment protocol we used was identical to a series of previous studies, in which we administered the same number of adult NPCs (10^6^ cells per animal) obtained via the same NPC isolation protocol to normolipidemic mice in this intraluminal MCAO model [9, 13–18]. In these earlier studies, NPCs consistently induced long-term cerebroprotection that was associated with reduced brain edema, blood–brain barrier permeability, brain leukocyte infiltration, microglia/macrophage activation and glial scar formation, when administered immediately or up to 6 h post-MCAO by our group [13, 14, 16–18]. In behavioral studies, improved recovery of motor-coordination deficits in Rotarod, tight rope and corner turn tests were found in NPC-treated mice compared with vehicle-treated mice that persisted up to 84 day post-MCAO [13, 17, 18]. While acute post-ischemic cerebroprotection, preservation of blood–brain barrier integrity and reduction of brain leukocyte infiltrates by NPCs were also noted in the peri-infarct tissue of hyperlipidemic MCAO mice, the lack of long-term effects of NPCs on structural tissue remodeling and functional neurological recovery indicate a state of compromised tissue recovery in the ischemic brains of hyperlipidemic mice.

Hyperlipidemia has profound effects on the cerebral microvasculature, inducing fatty streaks in brain arterioles within 6 weeks of Western diet exposure [21]. When exposed to focal cerebral ischemia induced by transient intraluminal MCAO, mice on Western diet exhibited increased activation of calpain-1/2 and matrix metalloproteinase-2/9, overactivation of RhoA and its guanine exchange factor leukemia-associated guanine exchange factor, and downregulation of the tight junction protein occludin in cerebral microvessels, resulting in increased blood–brain barrier permeability and brain swelling [21]. Besides, increased brain invasion by leukocytes and polymorphonuclear neutrophils associated with increased cerebral oxidative DNA damage were noted in the ischemic brains of hyperlipidemic mice on Western diet [22, 36]. Neutrophil depletion using anti-Ly6G antibody or neutrophil blockade using a selective CXC-motif chemokine receptor-2 (CXCR2) antagonist or a neutralizing anti-CXCR2 antibody markedly reduced brain leukocyte and neutrophil infiltrates, prevented the excessive oxidative stress and reversed the increased structural histological brain injury that was noted in hyperlipidemic compared with normolipidemic mice [22]. Post-ischemic neutrophil brain infiltrates pivotally contribute to ischemic brain injury and neurological impairments [35]. In mice exposed to intraluminal MCAO, neutrophil depletion using anti-Ly6G antibody reduced both infarct volume and motor-coordination impairments in Rotarod, tight rope and corner turn tests, while T cell depletion using anti-CD3 antibody decreased infarct volume without affecting motor-coordination impairments [35]. In line with these earlier studies, we observed increased infarct volume, blood–brain barrier permeability and brain leukocyte (including neutrophil, monocyte and T cell) infiltration in the ischemic brain of hyperlipidemic compared with normolipidemic mice. In addition to previous studies, we show that brain hemorrhage formation is increased in the ischemic brain of hyperlipidemic mice.

Lack of post-ischemic neurological recovery has previously been demonstrated after bone marrow-derived mesenchymal stromal cell (MSC) delivery in animal models for another vascular risk factor, that is, diabetes. In streptozotocin-induced type-1 diabetes with complete β-cell destruction [34], but not in type-2 diabetes with partial β-cell destruction [37], poor neurological recovery associated with aberrant angiogenesis, increased brain hemorrhages and increased animal deaths was noted in response to MSC administration in MCAO rats. Extracellular matrix breakdown is more severe in type-1 than type-2 diabetic rats, resulting in massive neuroinflammation characterized by destructive M1-macrophage infiltrates [34, 37, 38]. MSC treatment shifted brain macrophage infiltrates to a protective M2-phenotype in type-2, but not type-1 diabetic rats [34, 37, 38]. In our study, NPC administration reduced brain leukocyte infiltrates, namely, neutrophil (including activated neutrophils), monocyte (including inflammatory monocytes), T cell and NK cell infiltrates, in the ischemic brain tissue of hyperlipidemic MCAO mice, but increased brain hemorrhages in hyperlipidemic mice. Indeed, brain hemorrhage formation was significantly elevated in NPC-treated hyperlipidemic compared with NPC-treated normolipidemic mice. Microvascular thrombosis, which promotes ischemic brain injury in MCAO mice [32], was not influenced by NPC delivery, as was ICAM-1 abundance on endothelial cells. 3D LSFM analysis revealed that NPC delivery did not alter microvascular length or branching point density, but increased branch volume density and mean branch diameter in the peri-infarct striatum of hyperlipidemic mice. The latter findings may indicate a state of maladaptive vascular remodeling.

Strikingly, NPC administration induced pro-inflammatory responses in the peripheral blood of hyperlipidemic, but not normolipidemic mice. Hence, NPCs reduced post-ischemic blood CD4^+^ T cell, CD8^+^ T cell and B cell counts in normolipidemic MCAO mice, but increased monocyte, macrophage, CD4^+^ T cell, CD8^+^ T cell, B cell and NK cell counts in hyperlipidemic mice. The significance of peripheral blood leukocyte, namely, monocyte/macrophage and lymphocyte, responses for the progression of neuronal injury and neurological deficits has recently been outlined in neurodegenerative conditions, namely, in Alzheimer’s disease, Parkinson’s disease and amyotrophic lateral sclerosis models [39]. That NPC delivery induces anti-inflammatory responses in the blood of normolipidemic MCAO mice, but pro-inflammatory responses in hyperlipidemic mice, has so far not been shown. Type-1 diabetes is a severe risk factor associated with excessive blood glucose levels that strongly promotes inflammatory responses [34]. Yet, type-1 diabetes is a rare condition that in the U.S. affects ~ 0.5% of human subjects [40]. With a prevalence of 48.6% of U.S. citizens ≥ 40 years requiring lipid-lowering therapy [19], hyperlipidemia is ~ 100 times more common than type-1 diabetes in human subjects. From this perspective, the disturbed stroke recovery to NPC treatment in hyperlipidemic MCAO mice will deserve attention in the clinical translation of cell therapies. We do not have an explanation for the pro-inflammatory responses to NPCs in the blood of hyperlipidemic mice. The level of major histocompatibility complex (MHC) class I protein, a pro-inflammatory stimulus that displays antigens to cytotoxic T cells, on NPCs is generally low under physiological conditions, but may increase upon exposure to pro-inflammatory cytokines [41]. It is conceivable that hyperlipidemia-associated inflammation in the blood converted the NPCs to a phenotype that further elevated the inflammatory responses. Future studies will have to examine this question. Do we have to pause or even stop translational treatment efforts in light of almost half of the population carrying this detrimental risk factor? With the unprecedented observation that NPCs had pro-inflammatory actions in the blood of hyperlipidemic MCAO mice, but anti-inflammatory actions in normolipidemic mice, this study offers us a potential marker that might allow detecting detrimental effects of cell therapies in stroke patients. So far, blood leukocyte responses have not been studied systematically in early phase-2a clinical cell therapy trials, but we propose to include them as readouts in future studies.

Strengths of this study are that this study was fully blinded and rigorously powered, with effects of NPCs on neurological recovery analyzed in two consecutive studies with a sample size of in total 18–23 mice per group, which were evaluated in a clinically relevant neurological score, Rotarod and tight rope tests. We employed a 30 min MCAO model in this study that induces ischemic injury of the striatum and adjacent parietal cortex. The model is highly reproducible and has little animal dropouts. We decided against using a longer MCAO model in order to detect exacerbating effects of hyperlipidemia on ischemic injury and stroke outcome. This study used the same NPC preparation protocol, the same NPC delivery strategy and the same functional and structural readouts as our previous studies [9, 13–18]. Our findings highlight the necessity of rigorous investigations in vascular risk factor models to fully assess the long-term restorative effects of cell-based therapies. Without comprehensive studies in such models, the clinical potential of cell-based therapies cannot be definitely determined.

### Supplementary Information

**Additional file 1: Figure S1.** Gating strategies used for analyzing brain and peripheral blood leukocytes and leukocyte activation via flow cytometry. In two sets of studies, lymphoid and myeloid cells were examined. **Figure S2.** NPC administration does not influence microvascular ICAM-1 expression, but hemorrhage incidence is higher in NPC-treated hyperlipidemic compared to normolipidemic mice. (**A**) Intercellular adhesion molecule-1 (ICAM-1) abundance on ischemic microvessels assessed by immunohistochemistry and (**B**) hemorrhage incidence evaluated by diaminobenzidine staining of normolipidemic mice on normal diet and hyperlipidemic mice on Western diet, which were exposed to 30 min intraluminal middle cerebral artery occlusion (MCAO) and intravenously treated with vehicle (200 µl of 0.1 M phosphate-buffered saline [PBS]) or adult NPCs (10^6^ cells in 200 µl of 0.1 M PBS) immediately after reperfusion, followed by animal sacrifice at 48 h post-MCAO. Representative sections are shown. Data are medians (lines inside boxes)/means (crosses inside boxes) ± interquartile ranges with minimum/maximum values as whiskers. No significant group differences were noted (*n* = 12 mice for normal diet/vehicle, *n* = 12 for normal diet/NPC, *n* = 10 for Western diet/vehicle, *n* = 11 for Western diet/NPC). Scale bar, 50 μm. **Figure S3.** NPC administration does not influence neurological deficits in hyperlipidemic mice. Motor-coordination performance in (**A**) the Rotarod test and (**B**) the tight rope test, (**C**) body weight and (**D**) laser Doppler flow recordings above the core of the middle cerebral artery territory of mice on Western diet exposed to 30 min intraluminal MCAO, which were intravenously treated with vehicle or NPCs immediately after reperfusion (once only, dosing as before), followed by animal sacrifice after 56 days. Note that NPCs did not influence motor-coordination performance. For hyperlipidemic mice treated three times with NPCs see Fig. 6. Data are means ± S.D. values. No significant group differences were noted (*n* = 11 mice for Western diet/vehicle, *n* = 13 for Western diet/NPC). **Figure S4.** NPC delivery does not influence brain atrophy and glial scar formation in the chronic stroke phase in hyperlipidemic mice. (A) Brain atrophy, (B) striatal atrophy and (C) corpus callosum thickness assessed by cresyl violet staining, and (**D**) glial fibrillary acidic protein (GFAP) expression in the striatum assessed by immunohistochemistry in MCAO mice on Western diet, which were intravenously treated with vehicle or NPCs immediately after reperfusion (once only, dosing as before), followed by animal sacrifice after 56 days. For hyperlipidemic mice treated three times with NPCs see Fig. 7. Data are medians (lines inside boxes)/means (crosses inside boxes) ± interquartile ranges with minimum/maximum values as whiskers. No significant group differences were noted (*n* = 11 mice for Western diet/vehicle, *n* = 13 for Western diet/NPC). Scale bar, 1 mm [in (**A**–**C**)] and 50 μm [in (**D**)]. **Table S1.** Scoring of tight rope test performance. **Table S2.** Antibodies used for flow cytometry.

## Data Availability

The data used and/or analyzed in the current study are available from the corresponding author on reasonable request.

## Abbreviations

CD: Cluster of differentiation
CXCR: C–X–C motif chemokine receptor
ECi: Ethyl cinnamate
EGFP: Enhanced green fluorescence protein
GFAP: Glial fibrillary acidic protein
GP-Ibα: Glycoprotein-Ibα
Iba-1: Ionized calcium binding adaptor protein-1
ICAM-1: Intercellular adhesion molecule-1
LDF: Laser Doppler flow
MCAO: Middle cerebral artery occlusion
MHC: Major histocompatibility complex
MSC: Mesenchymal stromal cell
NeuN: Neuronal nuclei antigen
NK: Natural killer
NPC: Neural precursor cell
PBS: Phosphate-buffered saline
THF: Tetrahydrofuran
