# Supplementary material for: Neural precursor cell delivery induces acute post-ischemic cerebroprotection, but fails to promote long-term stroke recovery in hyperlipidemic mice due to mechanisms that include pro-inflammatory responses associated with brain hemorrhages
Source: J Neuroinflammation. 2023 Sep 15;20:210. doi: 10.1186/s12974-023-02894-8 (PMC10504699; doi:10.1186/s12974-023-02894-8)
Supplement: Supplementary file 1 — Additional file 1: Figure S1. Gating strategies used for analyzing brain and peripheral blood leukocytes and leukocyte activation via flow cytometry. In two sets of studies, lymphoid and myeloid cells were examined. Figure S2. NPC administration does not influence microvascular ICAM-1 expression, but hemorrhage incidence is higher in NPC-treated hyperlipidemic compared to normolipidemic mice. (A) Intercellular adhesion molecule-1 (ICAM-1) abundance on ischemic microvessels assessed by immunohistochemistry and (B) hemorrhage incidence evaluated by diaminobenzidine staining of normolipidemic mice on normal diet and hyperlipidemic mice on Western diet, which were exposed to 30 min intraluminal middle cerebral artery occlusion (MCAO) and intravenously treated with vehicle (200 µl of 0.1 M phosphate-buffered saline [PBS]) or adult NPCs (106 cells in 200 µl of 0.1 M PBS) immediately after reperfusion, followed by animal sacrifice at 48 h post-MCAO. Representative sections are shown. Data are medians (lines inside boxes)/means (crosses inside boxes) ± interquartile ranges with minimum/maximum values as whiskers. No significant group differences were noted (n = 12 mice for normal diet/vehicle, n = 12 for normal diet/NPC, n = 10 for Western diet/vehicle, n = 11 for Western diet/NPC). Scale bar, 50 μm. Figure S3. NPC administration does not influence neurological deficits in hyperlipidemic mice. Motor-coordination performance in (A) the Rotarod test and (B) the tight rope test, (C) body weight and (D) laser Doppler flow recordings above the core of the middle cerebral artery territory of mice on Western diet exposed to 30 min intraluminal MCAO, which were intravenously treated with vehicle or NPCs immediately after reperfusion (once only, dosing as before), followed by animal sacrifice after 56 days. Note that NPCs did not influence motor-coordination performance. For hyperlipidemic mice treated three times with NPCs see Fig. 6. Data are means ± S.D. values. No significant gr [file 12974_2023_2894_MOESM1_ESM.docx]

**Neural precursor cell delivery induces acute post-ischemic cerebroprotection, but fails to promote long-term stroke recovery in hyperlipidemic mice due to mechanisms that include pro-inflammatory responses associated with brain hemorrhages**

Dongpei Yin^1^, Chen Wang^1^, Yachao Qi^1^, Ya-Chao Wang^1,4^, Nina Hagemann^1^, Ayan Mohamud Yusuf^1^, Egor Dzyubenko^1^, Britta Kaltwasser^1^, Tobias Tertel^2^, Bernd Giebel^2^, Matthias Gunzer^3,5^, Aurel Popa-Wagner^1,6^, Thorsten R. Doeppner^1,7^, Dirk M. Hermann^1^

^1^Department of Neurology, ^2^Institute for Transfusion Medicine and ^3^Institute for Experimental Immunology and Imaging and Imaging Center Essen (IMCES), University Hospital Essen, University of Duisburg-Essen, Essen, Germany; ^4^Institute of Translational Medicine, The First Affiliated Hospital of Shenzhen University, Shenzhen Second People's Hospital, Shenzhen, China; ^5^Leibniz-Institut für Analytische Wissenschaften –ISAS– e.V., Dortmund, Germany, ^6^Center of Experimental and Clinical Medicine, University of Medicine and Pharmacy, Craiova, Romania; ^7^Department of Neurology, Justus-Liebig University Gießen, Gießen, Germany

Supplementary figures: 4

Supplementary tables: 2

Supplemental references: 1

**Corresponding author:** Prof. Dirk M. Hermann, MD, Department of Neurology, University Hospital Essen, Hufelandstr. 55, DE-45147 Essen, Germany; phone: +49-201-723-2814; fax: +49-201-723-5534; e-mail: [dirk.hermann@uk-essen.de](mailto:dirk.hermann@uk-essen.de)

**Additional file: Figures and tables**

**

**

**Figure S1.** Gating strategies used for analyzing brain and peripheral blood leukocytes and leukocyte activation via flow cytometry. In two sets of studies, lymphoid and myeloid cells were examined.


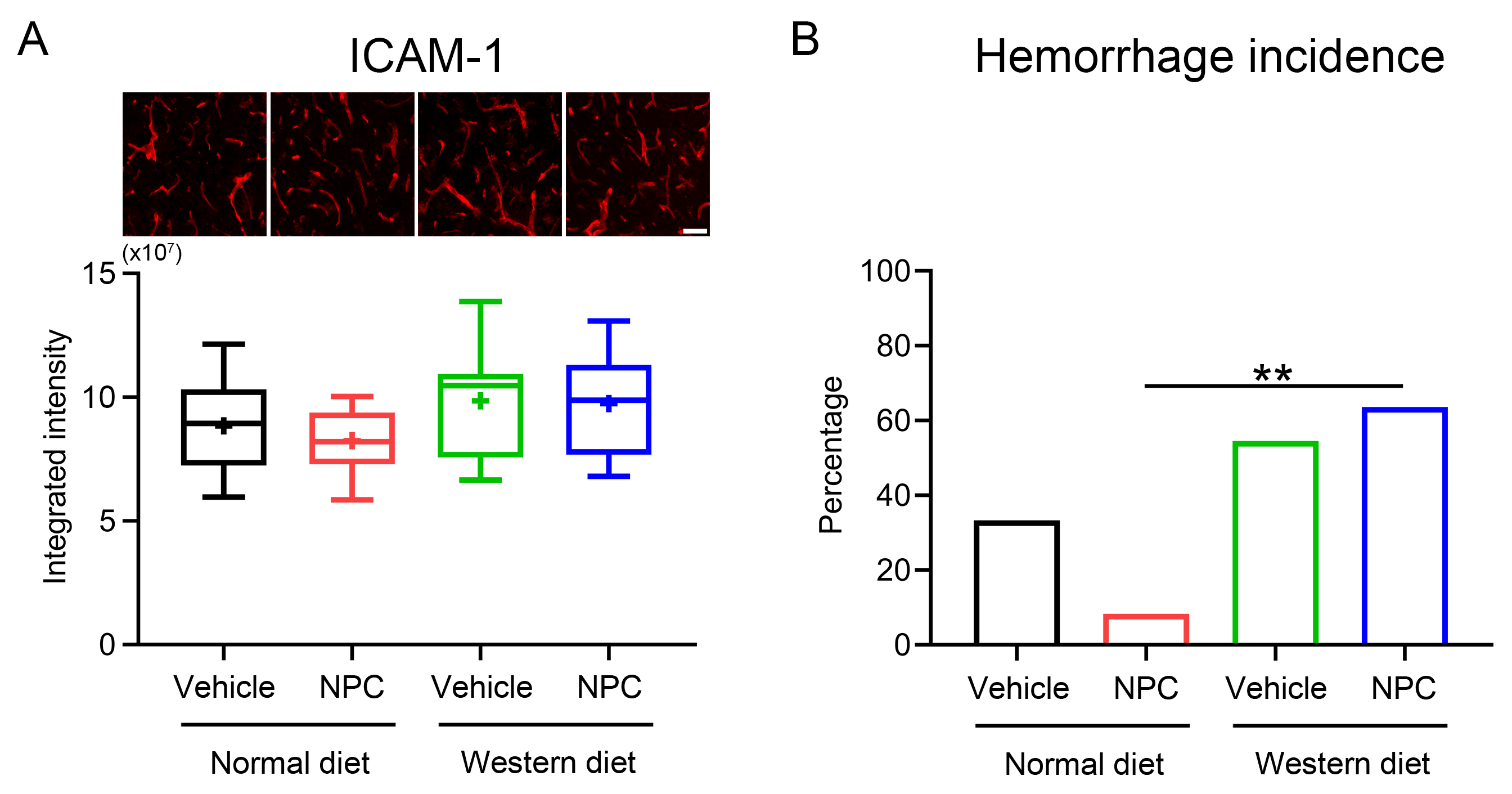


**Figure S2. NPC administration does not influence microvascular ICAM-1 expression, but hemorrhage incidence is higher in NPC-treated hyperlipidemic compared to normolipidemic mice.** (**A**) Intercellular adhesion molecule-1 (ICAM-1) abundance on ischemic microvessels assessed by immunohistochemistry and (**B**) hemorrhage incidence evaluated by diaminobenzidine staining of normolipidemic mice on normal diet and hyperlipidemic mice on Western diet, which were exposed to 30 min intraluminal middle cerebral artery occlusion (MCAO) and intravenously treated with vehicle (200 µl of 0.1 M phosphate-buffered saline [PBS]) or adult NPCs (10^6^ cells in 200 µl of 0.1 M PBS) immediately after reperfusion, followed by animal sacrifice at 48 hours post-MCAO. Representative sections are shown. Data are medians (lines inside boxes)/means (crosses inside boxes) ± interquartile ranges with minimum/maximum values as whiskers. No significant group differences were noted (n=12 mice for normal diet/ vehicle, n=12 for normal diet/ NPC, n=10 for Western diet/ vehicle, n=11 for Western diet/ NPC). Scale bar, 50 μm.


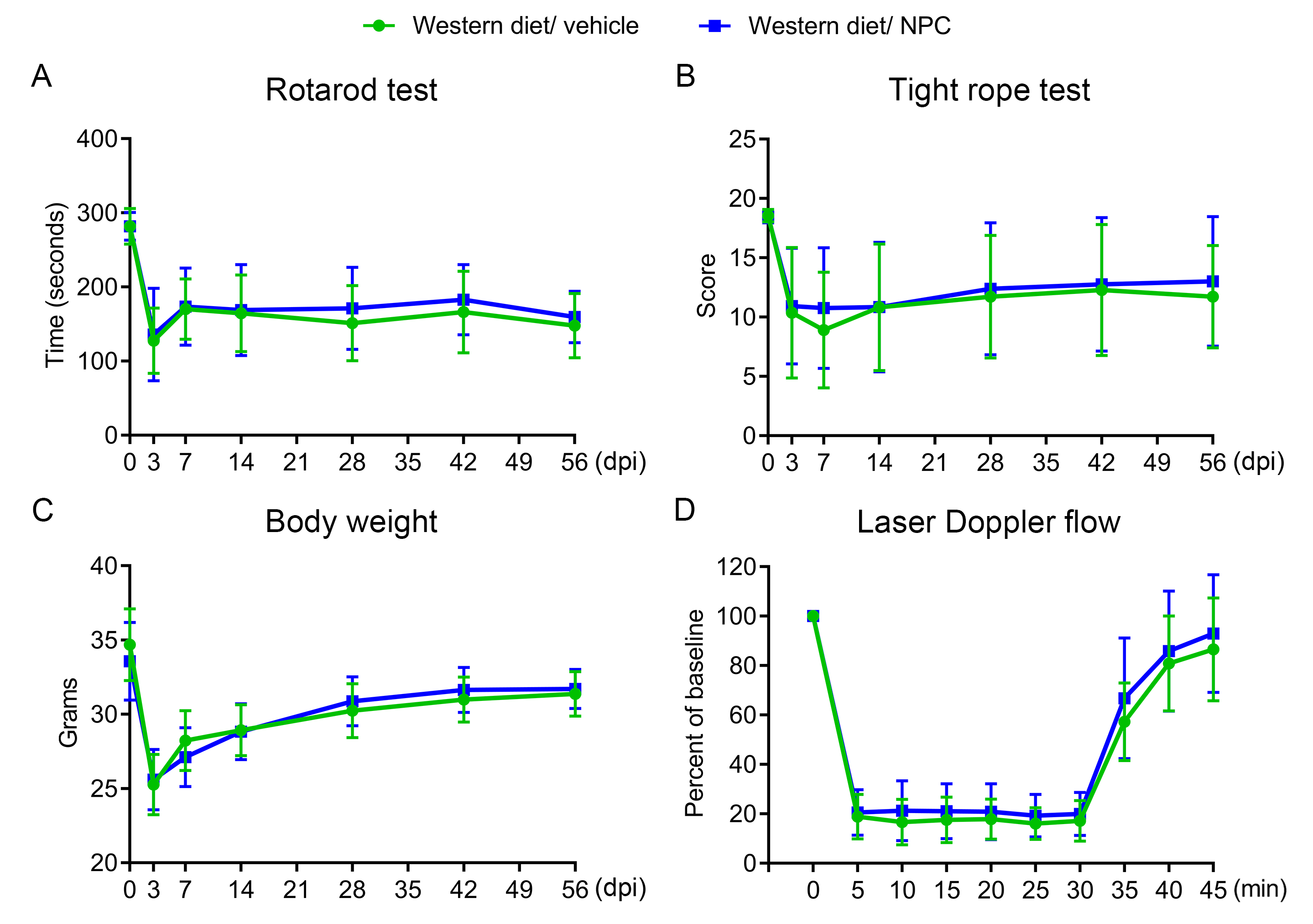


**Figure S3. NPC administration does not influence neurological deficits in hyperlipidemic mice.** Motor-coordination performance in (**A**) the Rotarod test and (**B**) the tight rope test, (**C**) body weight and (**D**) laser Doppler flow recordings above the core of the middle cerebral artery territory of mice on Western diet exposed to 30 min intraluminal MCAO, which were intravenously treated with vehicle or NPCs immediately after reperfusion (once only, dosing as before), followed by animal sacrifice after 56 days. Note that NPCs did not influence motor-coordination performance. For hyperlipidemic mice treated three times with NPCs see Figure 6. Data are means ± S.D. values. No significant group differences were noted (n=11 mice for Western diet/ vehicle, n=13 for Western diet/ NPC).

**
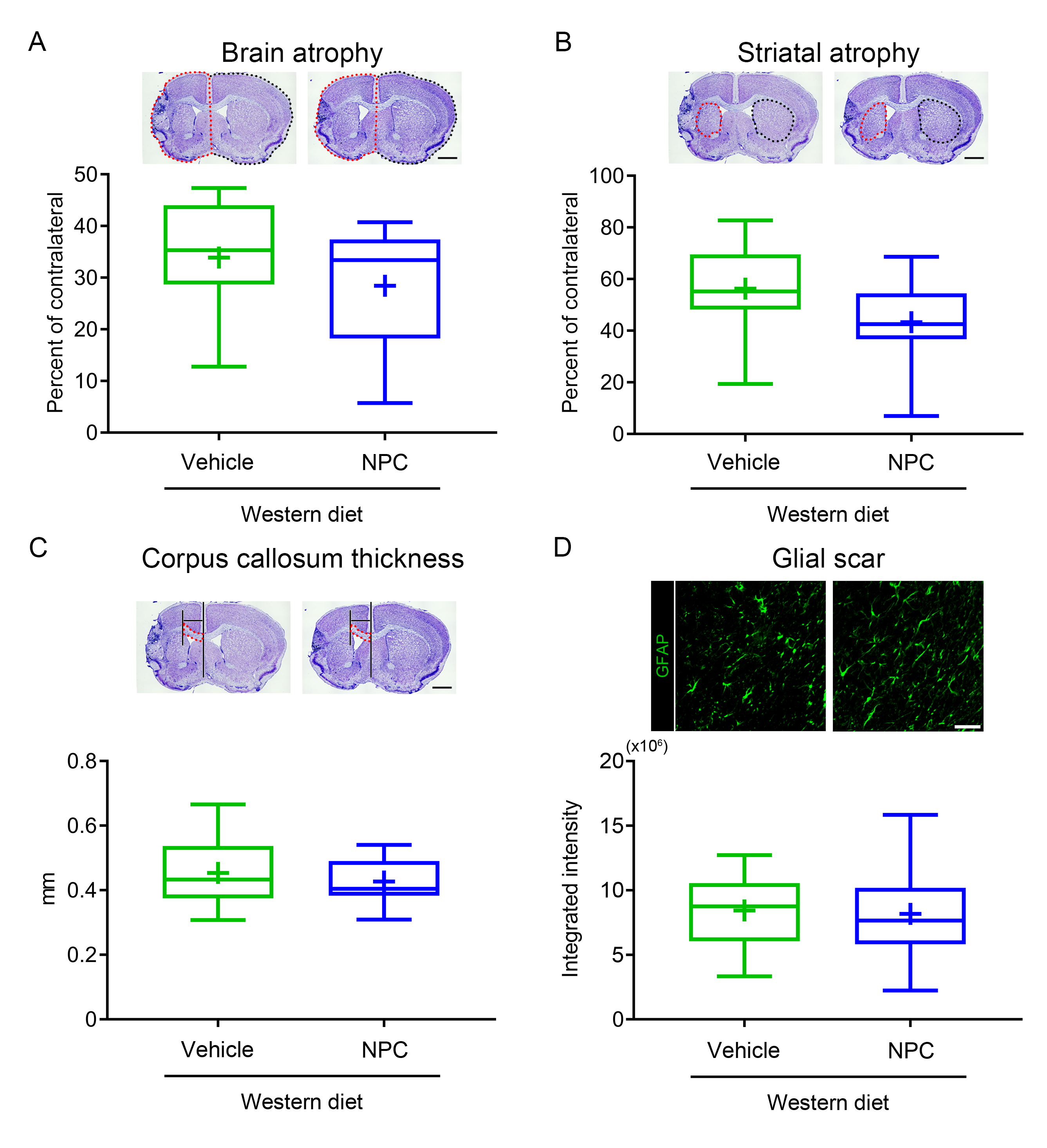
**

**Figure S4. NPC delivery does not influence brain atrophy and glial scar formation in the chronic stroke phase in hyperlipidemic mice.** (**A**) Brain atrophy, (**B**) striatal atrophy and (**C**) corpus callosum thickness assessed by cresyl violet staining, and (**D**) glial fibrillary acidic protein (GFAP) expression in the striatum assessed by immunohistochemistry in MCAO mice on Western diet, which were intravenously treated with vehicle or NPCs immediately after reperfusion (once only, dosing as before), followed by animal sacrifice after 56 days. For hyperlipidemic mice treated three times with NPCs see Figure 7. Data are medians (lines inside boxes)/means (crosses inside boxes) ± interquartile ranges with minimum/maximum values as whiskers. No significant group differences were noted (n=11 mice for Western diet/ vehicle, n=13 for Western diet/ NPC). Scale bar, 1 mm (in (**A-C**)) and 50 μm (in (**D**)).

**Table S1.** Scoring of tight rope test performance

| Score | Time (second) | Platform arrival |
| --- | --- | --- |
| 20 | 1-6 | + |
| 19 | 7-12 | + |
| 18 | 13-18 | + |
| 17 | 19-24 | + |
| 16 | 25-30 | + |
| 15 | 31-36 | + |
| 14 | 37-42 | + |
| 13 | 43-48 | + |
| 12 | 49-54 | + |
| 11 | 55-60 | + |
| 10 | 55-60 | - |
| 9 | 49-54 | - |
| 8 | 43-48 | - |
| 7 | 37-42 | - |
| 6 | 31-36 | - |
| 5 | 25-30 | - |
| 4 | 19-24 | - |
| 3 | 13-18 | - |
| 2 | 7-12 | - |
| 1 | 1-6 | - |
| 0 | 0 | - |

Tight rope test results were assessed according to the time on the rope and platform arrival, ‘‘+’’ for arrival and ‘‘-’’ for non-arrival. The scores ranged from 0 (minimum) to 20 (maximum).[1]

**Table S2.** Antibodies used for flow cytometry

| Antigen | Conjugate | Host/isotype | Clone | Supplier |
| --- | --- | --- | --- | --- |
| Mouse CD45 | Pacific blue | Rat IgG2b, kappa | 30F11 | BioLegend |
| Mouse CD45 | BV 605 | Rat IgG2b, kappa | 30F11 | BioLegend |
| Mouse Ly6G | Phycoerythrin (PE) | Rat IgG2a, kappa | 1A8 | BioLegend |
| Mouse CXCR2 | Peridinin-chlorophyll  (PerCP)-Cy5.5 | Rat IgG2a, kappa | SA044G4 | BioLegend |
| Mouse CD62L | eFluor 450 | Rat IgG2a, kappa | MEL-14 | eBioscience |
| Mouse Ly6C | Fluorescein isothiocyanate (FITC) | Rat IgM, kappa | AL21 | BD Biosciences |
| Mouse CD11b | PerCP-Cy5.5 | Rat IgG2b, kappa | M1/70 | BioLegend |
| Mouse CD115 | PE-Cy7 | Rat IgG2a, kappa | AFS98 | eBioscience |
| Mouse CD3ε | Alexa Fluor 647 | Hamster IgG | 145-2C11 | BioLegend |
| Mouse CD4 | BV 605 | Rat IgG2a, kappa | RM4-5 | BD Biosciences |
| Mouse CD8 | BV 786 | Rat IgG2a, kappa | 53-6.7 | BD Biosciences |
| Mouse B220 | PE | Rat IgG2a, kappa | RA3-6B2 | BD Biosciences |
| Mouse CD19 | APC | Rat IgG2a, kappa | 1D3 | BioLegend |
| Mouse NK-1.1 | FITC | Rat IgG2a, kappa | PK136 | BD Biosciences |
| Mouse CD69 | PE-Cy7 | Hamster IgG | H1.2F3 | BioLegend |

**Additional reference**

1. Doeppner TR, Bretschneider E, Doehring M, Segura I, Senturk A, Acker-Palmer A, Hasan MR, ElAli A, Hermann DM, Bahr M: Enhancement of endogenous neurogenesis in ephrin-B3 deficient mice after transient focal cerebral ischemia. *Acta Neuropathol* 2011, 122:429-442.
